# Dataset of mathematics learning and assessment of higher education students using the MathE platform

**DOI:** 10.1016/j.dib.2024.110236

**Published:** 2024-02-28

**Authors:** Beatriz Flamia Azevedo, Maria F. Pacheco, Florbela P. Fernandes, Ana I. Pereira

**Affiliations:** a Research Centre in Digitalization and Intelligent Robotics (CeDRI), Instituto Politécnico de Bragança, Bragança 5300-253, Portugal; b Laboratório Associado para a Sustentabilidade e Tecnologia em Regiões de Montanha (SusTEC), Instituto Politécnico de Bragança, Bragança 5300-253, Portugal

**Keywords:** Active learning, Collaborative learning, E-learning, Educational technology, Student engagement, Learning analytics, Adaptive learning, math

## Abstract

Higher education institutions are promoting the adoption of innovative methodologies and instructional approaches to engage and promote personalized learning paths to their students. Several strategies based on gamification, artificial intelligence, and data mining are adopted to create an interactive educational setting centred around students. Within this personalized learning environment, there is a notable boost in student engagement and enhanced educational outcomes. The MathE platform, an online educational system introduced in 2019, is specifically crafted to support students tackling difficulties in comprehending higher-education-level mathematics or those aspiring to deepen their understanding of diverse mathematical topics — all at their own pace. The MathE platform provides multiple-choice questions, categorized under topics and subtopics, aligning with the content taught in higher education courses. Accessible to students worldwide, the platform enables them to train their mathematical skills through these resources. When the students log in to the training area of the platform, they choose a topic to study and specify whether they prefer basic or advanced questions. The platform then selects a set of seven multiple-choice questions from the available ones under the chosen topic and generates a test for the student. After completing and submitting the test, the answers are recorded and stored on the platform. This paper describes the data stored in the MathE platform, focusing on the 9546 answers to 833 questions, provided by 372 students from 8 countries who use the platform to practice their skills using the questions (and other resources) available on the platform. The information in this paper will help research about active learning tools to support the improvement of future education, especially at higher educational level. Furthermore, these data are valuable for understanding student learning patterns, assessing platform efficacy, gaining a global perspective on mathematics education, and contributing to the advancement of active learning tools for higher education.

Specifications Table

**Table:** 

Subject	Data Engineering
Specific subject area	*Assessment of higher education students in Math topics.*
Data format	Raw
Type of data	Table
Data collection	The dataset was collected online by the MathE platform system. It includes the answers collected by the MathE platform, for 14 topics and 24 subtopics of mathematical contents taught in higher education courses and are available on the MathE platform. Moreover, each question is associated with a set of keywords that describe the question main contents. The answers are described in terms of correct (0) or incorrect (1), and they were provided by 372 students identified by Identification Number (ID). Besides, it is available the information about the country that each students belongs. Thus, in this dataset there are 9546 instances available.
Data source location	The dataset was provided by higher education students from eight countries: Portugal, Lithuania, Italy, Ireland, Romania, Russia, Spain, and Slovenia.
Data accessibility	Repository name: Instituto Politécnico de Bragança – Data Repository Data identification number: https://doi.org/10.34620/dadosipb/PW3OWY Direct URL to access data: https://dados.ipb.pt//dataset.xhtml?persistentId=doi:10.34620/dadosipb/PW3OWY
Related research article	Azevedo, B.F., Pereira, A.I., Fernandes, F.P., Pacheco, M.F.: Mathematics learning and assessment using MathE platform: A case study. Education and Information Technologies (2021). https://doi.org/10.1007/s10639-021-10669-y

## 1 Value of the Data

• The dataset provides valuable insights into how students from diverse backgrounds engage with and respond to mathematics questions. This information can help educators and researchers understand learning behaviors, preferences, and challenges, as well as supporting students with different levels of proficiency in higher-level Mathematics.
• The data encompasses responses from students from eight countries, offering a global perspective on learning trends and challenges in Mathematics education. This broad view allows for cross-cultural comparisons and identifies patterns that may be specific to certain regions or applicable universally.
• The data shed light on the effectiveness of personalized learning, as students can make choices on topics, question difficulty levels, and pace of study. Understanding how students navigate and benefit from personalized learning experiences contributes to the broader conversation on educational technology and tailored learning approaches.
• The information gathered is crucial for investigations into active learning tools. It provides a basis for assessing the impact of such tools on student engagement, knowledge retention, and overall learning outcomes. This, in turn, supports the ongoing improvement of active learning strategies in higher education.
• The dataset allows for the identification of specific challenges that students face in understanding college-level mathematics. By analyzing common misconceptions, areas of difficulty, and patterns of errors, educators can tailor instructional strategies to address these challenges more effectively.

## 2 Background

Active learning tools encompass various strategies that go beyond passive listening to lectures, engaging students in activities that promote discovery, information processing, and application tasks [5,8]. Case study research has consistently demonstrated that the implementation of active teaching strategies correlates with increased lecturer attendance, heightened engagement, the development of expert attitudes among students towards the subject, and enhanced overall student abilities [5,6,9,10]. It is important, however, to recognize the significance of traditional lecturing, emphasizing that active learning should always be complemented by well-defined content and clear learning objectives [8].

Individuals differ in their profiles of strengths and weaknesses across different types of intelligence, with educational and professional success hinging on the capacity to bolster strengths and offset weaknesses [7]. The challenge arises from the impossibility of teaching effectively to every student's unique learning style. Customized education based on active learning strategies has the potential to revolutionize the way we approach teaching and learning. By recognizing the unique needs and abilities of individual students, we can help to create a more equitable and effective education.

## 3 Data Description

MathE is a pioneering collaborative e-learning platform that enhances the usersʼ mathematical learning processes in higher education. Its core objective is to cultivate virtual learning and foster knowledge exchange [1].

MathE goals enhance the quality of teaching, learning, and assessment methods in higher education Mathematics content. This platform is a non-commercial tool, entirely free for those interested in improving their knowledge and understanding of Mathematics. MathE has been online since 2019 at https://mathe.pixel-online.org. The platform comprises two main sections: the *MathE Library* and *Students Assessment*. The library offers a collection of videos and written materials to support the acquisition of knowledge; in the *Students Assessment* section, there is a set of multi-choice questions to provide the student training assessment to test if a specific topic that he/she enrolled in is already well understood.

Moreover, MathE provides an associated YouTube channel (available at https://www.youtube.com/@matheproject4778) with a collection of videos linked to some topic or subtopic available on the platform. For more details about the platform, refer to [1], [2], [3].

The dataset [4] is composed of the data collected on the platform system from January 2019 until December 2023, and it is described in terms of 9546 instances and 8 features, as described below:

• **Student ID:** is a numeric identification (ID) assigned to the student at the moment that he/she registers on the platform. The dataset contemplates 372 distinct students.
• **Student Country:** provides information about the country where the student is studying. The dataset comprises students from 8 countries, as also described in Table 3: Portugal, Lithuania, Italy, Ireland, Romania, Russia, Spain, and Slovenia.
• **ID question:** is a numeric value that identifies each question. The dataset is composed of the answers to 833 distinct questions.
• **Type of answer:** it describes the type of answer provided by the student for each question, in terms of correct answer (1) or incorrect answer (0).
• **Question level:** it defines the level of the question answered. It was considered two levels, basic and advanced. This level was identified by the lecture that submit the question.
• **Topic:** it describes in which topic belongs the question answered, among the ones presented in Table 1.

**Table 1:** Data per topics and subtopics.

Topic	Number of answers	Subtopic	Number of answers
Linear Algebra	5726	Eigenvectors and Eigenvalues	130
		Linear Systems	420
		Linear Transformation	2127
		Matrices and Determinants	300
		Vector Spaces	2749
Fundamental Mathematics	818	Algebraic Expressions, Equations, and Inequalities	496
		Elementary Geometry	322
Graph Theory	55	Graph Theory	55
Differentiation	579	Derivatives	317
		Partial Differentiation	262
Integration	144	Integration Techniques	111
		Definite Integrals	18
		Double Integrals	15
Analytic Geometry	358	Analytic Geometry	358
Complex Numbers	592	Complex Numbers	592
Differential Equations	108	Differential Equations	108
Statistic	340	Statistic	340
Real Functions of a Single Variable	164	Domain, Image, and Graphics	107
		Limits and Continuity	57
Probability	128	Probability	128
Optimization	182	Linear Optimization	56
		Nonlinear Optimization	126
Set Theory	42	Set Theory	42
Numerical Methods	310	Numerical Methods	310
• **Subtopic:** it describes in which subtopic belongs the question answered, among the ones presented in Table 1.
• **Question keywords:** it defines a set of keywords that describe the main contents involved in the question and selected by the lecturer that submits the question.

Table 2 describes the number of obtained answers considering the type of answer, if it is correct or incorrect, and the level of the question, basic or advanced.

**Table 2:** Answers per type and level.

Type of answer	Basic level answers	Advanced level answers
Correct	3617	853
Incorrect	4227	849

The platform is currently being used by a significant number of users. However, some of these students use the platform only to consult the materials, not to answer the questions. Thus, the dataset [4] contemplates the answers provided by students from 8 countries, as described in Table 3.

**Table 3:** Data per countries.

Country	Number of students	Number of answers
Portugal	245	5495
Ireland	13	300
Italy	50	1358
Lithuania	56	1443
Romania	1	28
Russia	1	107
Spain	3	60
Slovenia	3	755

Concerning the keywords, the dataset consists of 194 keywords distributed across the topics and subtopics within the platform. These keywords are intended to elucidate about the primary content covered in the questions and assist users in effortlessly locating the materials they wish to explore, according to specific contents.

## 4 Experimental Design, Materials and Methods

MathE is a collaborative platform maintained by a group of lecturers who voluntarily create, insert, and review questions and materials to contribute to the platformʼs content. Thus, lecturers are tasked with contributing questions and materials to support the platform. When they wish to add a question, they are required to select a topic, subtopic, and level for the question in the system. Additionally, they must associate written materials or videos that aid in the student learning process, specifically related to the content covered in that particular question. Subsequently, the question and associated materials undergo a review process by a lecturer reviewer, who determines whether the content is approved or rejected for inclusion on the platform. If approved, the question becomes part of the available set for students to attempt, and the associated materials are incorporated into the MathE library. The library can be accessed directly, or the materials may be suggested to students after completing a test. Lecturers have the flexibility to introduce new questions as needed. Consequently, the dataset does not exhibit a balanced distribution of answers per question. In other words, a recently added question typically has fewer answers compared to an older one.

On the other hand, within the *Students Assessment* section, students can improve their knowledge by engaging with the questions provided on the platform. To access these questions, students must specify the topic, subtopic, and difficulty level (basic or advanced) they wish to focus on for their practice. With this information, the platform generates a test comprising seven randomly chosen questions from the MathE data system, aligning with the students' specified criteria. Upon completing the test, the platform provides feedback by indicating which questions were answered correctly and which ones were answered incorrectly. For incorrectly answered questions, the platform suggests associated materials (videos, written notes, solved exercises) to guide the students in moving forward in their learning path. Additionally, the platform records information such as student ID, the questions attempted, and the types of answer in its database.

**Fig. 1:**
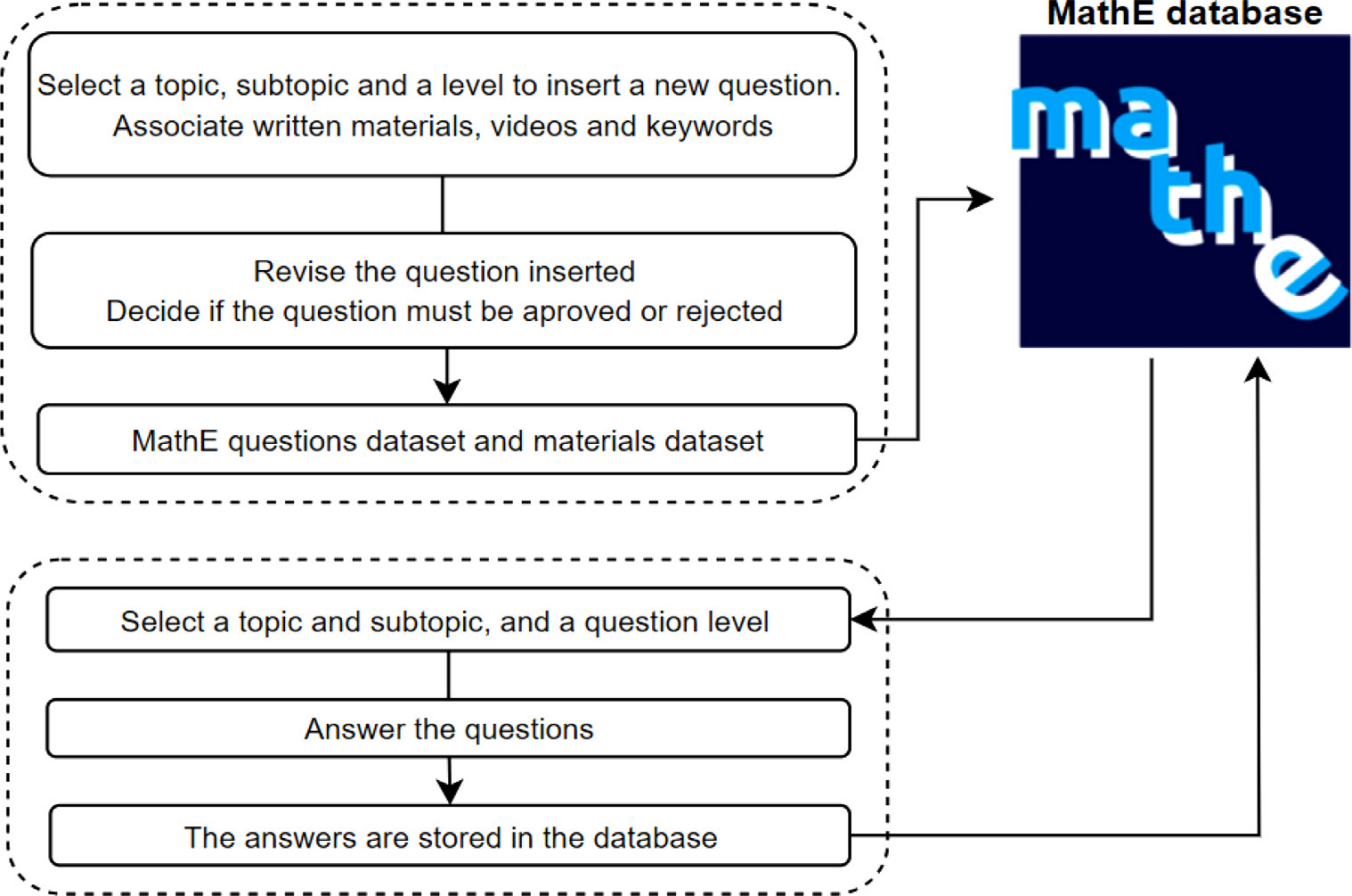
MathE data system.

In this manner, by cross-referencing the information related to both the questions and the students, the dataset presented in [4] is derived. Fig. 1 illustrates the MathE data system.

## Limitations

The items described below do not strictly constitute limitations of the database, but should be considered carefully, depending on the type of analysis one wishes to perform:

• Some questions have been answered many times, while others have been answered few or only once.
• The number of answers to basic questions is much higher than the number of answers to advanced questions.
• The dataset is not balanced in term of country: the majority of answers were provided by students from Portugal, Lithuania, and Italy.

## Ethics Statement

The authors have read and follow the ethical requirements for publication in Data in Brief and confirm that the current work does not involve human subjects, animal experiments, or any data collected from social media platforms.

## CRediT authorship contribution statement

**Beatriz Flamia Azevedo:** Conceptualization, Methodology, Data curation, Software, Investigation, Writing – original draft, Writing – review & editing. **Maria F. Pacheco:** Conceptualization, Visualization, Supervision, Investigation, Project administration, Writing – review & editing. **Florbela P. Fernandes:** Conceptualization, Visualization, Supervision, Investigation, Project administration, Writing – review & editing. **Ana I. Pereira:** Conceptualization, Visualization, Supervision, Validation, Investigation, Funding acquisition, Project administration, Writing – review & editing.

## Data Availability

Dataset for Assessing Mathematics Learning in Higher Education (Original data) (Instituto Politécnico de Bragança – Data Repository).
